# Individual Differences in Global Cognition Modulate the Effect of Motor-Relatedness on Object Naming in Healthy Older Adults

**DOI:** 10.3390/bs15030336

**Published:** 2025-03-10

**Authors:** Yang Xiao, Yanping Dong

**Affiliations:** Language Processing and Development Lab, School of International Studies, Zhejiang University, Hangzhou 310058, China; xiaoyang2627@zju.edu.cn

**Keywords:** motor-relatedness, older adults, embodied cognition, timed picture naming, global cognition, Montreal Cognitive Assessment (MoCA)

## Abstract

Lexical retrieval difficulty is a common daily complaint among older adults. Recent evidence suggests that older adults name motor-related nouns (e.g., knife) more accurately than non-motor nouns (e.g., steak). However, it remains unclear whether this motor-relatedness effect can reduce older adults’ object naming latency (a potentially more sensitive measure of word retrieval than accuracy) and how it may be modulated by individual differences (e.g., age and global cognition). Therefore, we recruited a large number of older adults to complete a Chinese version of the Montreal Cognitive Assessment (MoCA) and a timed picture-naming task, and we explored the two remaining issues with data from 76 community-dwelling older adults (65–81 years old), excluding participants with possible AD. Linear mixed-effects analysis revealed a main effect of motor-relatedness on naming latency in older adults and a significant interaction with the MoCA score after controlling for a number of stimulus-related factors (i.e., age of acquisition, familiarity, name agreement, and visual complexity) and participant-related factors (i.e., gender and education) as covariates, but age showed neither a main effect nor a significant interaction with motor-relatedness. Further simple slope analysis showed that older adults were faster at naming objects with high motor-relatedness and that older adults with low MoCA scores benefited more from the motor-relatedness effect. These findings suggest that motor-relatedness may compensate for the normal course of cognitive ageing in older adults. Implications for the motor-relatedness effect were discussed.

## 1. Introduction

Lexical retrieval difficulty is a common daily complaint among older adults. Recent evidence suggests that older adults name motor nouns more accurately than non-motor nouns (Reifegerste et al., 2021). The finding that motor-relatedness (the extent to which a word is associated with specific human body movements; Reifegerste et al., 2021; see also Herrera et al., 2012) is a facilitating factor for older adults’ lexical retrieval is consistent with embodied cognition principles, which predict that motor-related words engage distinct sensorimotor networks during lexical retrieval, activating areas involved in action. This activation is hypothesized to create multimodal memory traces that provide alternative retrieval pathways through motor circuitry and thereby may facilitate lexical retrieval through complementary semantic representations (Kiefer & Pulvermüller, 2012; Miklashevsky et al., 2024; Reifegerste et al., 2021; Yang, 2014). However, it remains unclear whether the motor-relatedness effect can reduce older adults’ object naming latency (a potentially more sensitive measure of lexical retrieval than accuracy; Gordon & Kindred, 2011; Wierenga et al., 2008). Furthermore, to our knowledge, the influence of age and global cognition on the presence of the motor-relatedness facilitation effect has not been explicitly investigated in healthy older adults, i.e., older adults with no diagnosed brain lesions, a history of stroke, or other overt medical conditions known to contribute directly to pathological cognitive impairment and without severe cognitive impairment (MoCA score ≥ 18, a cutoff chosen to exclude individuals with severe cognitive impairment while allowing for the inclusion of those with potential mild cognitive changes that may occur in normal aging; Mellor et al., 2016; Song et al., 2023).

Global cognition, also known as the general cognitive ability, includes various cognitive functions such as memory, attention, language, and abstraction (Hobson, 2015; Nasreddine et al., 2005). It can be measured by the Montreal Cognitive Assessment (MoCA), which is widely used by researchers and clinicians worldwide to assess global cognition in older adults (Mellor et al., 2016). Global cognition is critical in tasks measuring lexical retrieval (e.g., picture-naming tasks), because cognitive processes are required for efficient lexical retrieval. Such attempts at investigating the role of age and global cognition on the motor-relatedness effect may help clarify the details of when and how the motor-relatedness of objects may facilitate language processing (i.e., lexical retrieval) and provide evidence for the development of embodied cognition into a lifespan theory. By integrating multiple dimensions of individual differences (i.e., age, gender, education, and global cognition) into the analysis of motor-relatedness effects in older adults, the present study offers a novel and holistic approach to understanding this complex relationship.

The timed picture-naming task is widely used to measure lexical retrieval performance (Szekely et al., 2005), and naming latency (the time interval between the presentation of a visual stimulus and the initiation of the verbal response) is potentially more sensitive than accuracy to vary as a function of stimulus-related characteristics (e.g., age of acquisition, familiarity, and visual complexity) and individual characteristics (e.g., age, gender, education, and global cognition) (Gordon & Kindred, 2011). Among these stimulus-related factors, motor-relatedness has rarely been investigated in healthy older adults, although it has been investigated in young adults (Guérard et al., 2015; Salmon et al., 2010), brain-damaged populations, and patients with neurodegenerative diseases (Bowles et al., 1987; Druks et al., 2006; Herrera et al., 2012; Johari et al., 2019; Lai & Lin, 2013). Moreover, for researchers in cognitive psychology, these individual differences in naming latency can provide profound insights into the unique cognitive strategies that individuals employ during lexical retrieval. This will help to further our understanding of how the human mind processes language. In addition, understanding cognitive compensation mechanisms through this research can inform early detection of pathological aging using picture naming as a screening tool, and it also explores the feasibility of motor-based interventions for lexical retrieval in older adults, thus having significant societal relevance.

### 1.1. Motor-Relatedness Effects

Although the term “motor-relatedness” has rarely been directly used in previous studies, the issue of motor–language association has been widely discussed in terms of noun–verb distinction, semantic category distinction (e.g., different processing of living things and artifacts), and manipulability. Motor-relatedness first gained attention as the noun–verb distinction, and evidence suggests that verb processing may be more demanding than noun processing (Zhang et al., 2018). Multiple studies based on the picture-naming paradigm (i.e., action naming and object naming) have found that people may suffer from selective deficits in verb naming or in noun naming (e.g., Almor et al., 2009; Zhang et al., 2018). For example, patients with Parkinson’s disease (PD; a neurodegenerative disorder associated with motor problems) have more difficulty in naming action verbs than in naming object nouns (Rodríguez-Ferreiro et al., 2009). Evidence from other populations has shown a processing disadvantage for verbs, that is, longer naming latencies and higher error rates in healthy participants (Zhang et al., 2018), later learning in children (Waxman et al., 2013), and vulnerability to brain damage (Mätzig et al., 2009). They attribute the processing disadvantage to differences in grammatical complexity (i.e., verbs in many languages have complex inflectional systems in number, tense, aspect, mood, and gender).

Recently, researchers have recognized that action–verb or object–noun stimuli may be an important confounding factor and suggested that future motor–language association studies should consider finer-grained categories within the action and object sets (Zhang et al., 2018). Within the verb category, Herrera et al. (2012) used the action naming task and found that PD patients retrieved poorer results for high-motor-related actions than for low-motor-related actions. Similarly, within the noun category, Johari et al. (2019) used the object naming task and found that PD patients were more impaired in naming manipulated objects than non-manipulated objects. In fact, object manipulability (the degree to which an object can be manipulated by a person’s hand) has been considered a predictor of object naming latency (Ni et al., 2019). However, findings on the effect of manipulability were quite mixed. For example, some studies found that people named objects that were easier to grasp more rapidly (Guérard et al., 2015; Lorenzoni et al., 2018); however, Ni et al. (2019) found that object manipulability did not contribute to naming latencies.

The mixed results are probably due to two types of factors: the different definitions of manipulability (see Glenberg et al., 2008) they use, and some individual differences that may mediate the effect of manipulability on language processing (Ni et al., 2019; see also Glenberg et al., 2008; Helbig et al., 2010; Yang, 2014). First, the null result in Ni et al. (2019) is probably due to the narrow scope of their definition of manipulability. In addition to grasping, previous studies have included other motor dimensions of objects (e.g., pantomime and move; Guérard et al., 2015; Lorenzoni et al., 2018) and have shown facilitation effects on naming latency after controlling for covariates such as age of acquisition (AoA) and familiarity (Lorenzoni et al., 2018). Second, some individual difference factors may explain the mixed findings. For example, there is evidence that individual motor experiences initiated prior to language tasks (e.g., motor preparation, motor execution, and motor imagery, motor training and motor expertise; Yang, 2014) may influence language performance (see also Glenberg et al., 2008; Helbig et al., 2010).

To sum up, although previous studies on motor–language association suggest a facilitation effect of motor-relatedness in lexical retrieval, several factors preclude a consistent conclusion (e.g., different cognitive demands of verb and noun processing, narrow definition of manipulability, uncontrolled individual factors). Therefore, it may be better to address the issue within the verb/noun category (e.g., to contrast the different processing of high- and low-motor-related objects), to extend the motor dimension from hand-related to body-related motor, and, most importantly, to take more factors of individual difference into consideration. Given the mixed results and limitations of previous studies on motor-relatedness, it is important to examine how cognitive differences may interact with this factor, which is the focus of the next section.

### 1.2. Individual Differences in Gognition and Motor-Relatedness Effects

The extant literature also hints at the possibility that cognitive differences may influence the presence of motor-relatedness effects and modulate the facilitation effect of motor-relatedness in lexical retrieval. As discussed above, verb processing is more demanding than noun processing (Zhang et al., 2018), but evidence from healthy older adults and patients with Alzheimer’s disease (AD) has shown a trend towards a processing advantage for verbs over nouns. First, there is evidence that verb naming was relatively better preserved than noun naming in healthy aging (Barresi et al., 2000; see also Mackay et al., 2002). Second, there are conflicting findings in patients with Alzheimer’s disease (AD; a progressive form of dementia that affects memory, thinking, language, and behavior). Most studies of AD patients show that their ability to name actions is better preserved than their ability to name objects (e.g., Bowles et al., 1987; Druks et al., 2006; Lai & Lin, 2013), while some other studies have found the opposite pattern, i.e., that AD patients are better at naming objects than actions (but the distinction was not significant; Cotelli et al., 2012), or they have found no difference between object naming and action naming (Cappa, 2008; see also Lai & Lin, 2013). Therefore, grammatical complexity and cognitive demands are not sufficient to explain the processing distinction between nouns and verbs, because despite the relatively high processing demands of verbs, the noun processing advantage is not consistently present in populations with relatively poor global cognition performance (i.e., older adults and AD patients).

Reifegerste et al. (2021) investigated the interaction between motor-relatedness and aging in lexical processing, providing evidence for the modulatory role of global cognition in the motor-relatedness effect. Their study involved 49 native English speakers (age range: 18–72 years; *M* = 41.8, SD = 17.3) who completed an untimed picture-naming task with 64 stimuli (32 motor-related, e.g., shovel; 32 non-motor-related, e.g., panda). Using mixed-effects logistic regression, they found a significant main effect of motor-relatedness and an interaction between age and motor-relatedness. Specifically, older adults showed lower accuracy for non-motor nouns compared to younger adults, but no age-related decline was observed for motor nouns. These findings suggest that motor-relatedness may mitigate age-related declines in lexical retrieval.

However, the study has several limitations. First, the non-motor stimuli were exclusively animals, potentially confounding the motor-relatedness effect with the living/non-living semantic category effect. Second, naming latencies—a critical measure of lexical retrieval processes—were not recorded, limiting the insight into age-related retrieval difficulties (Wierenga et al., 2008). Third, stimulus-related factors (e.g., visual complexity) that may influence naming performance, especially in older adults, were not controlled (Hamberger et al., 2022). Fourth, participant-related factors, such as global cognition, education, and gender, were not examined, despite evidence suggesting their influence on naming performance and the motor-relatedness effect (Bidet-Ildei et al., 2020; Goral et al., 2007; Mellor et al., 2016). For example, Goral et al. (2007) found gender differences in action and object naming, with females outperforming males in action naming.

Although some studies suggest no motor-relatedness effect for older adults (Bidet-Ildei et al., 2020; Miklashevsky et al., 2024), these used tasks that require less semantic access than picture naming, such as lexical decision and reading aloud (Miklashevsky et al., 2024). Reifegerste et al. (2021) conducted lexical decision experiments with Dutch verbs and German nouns and found motor-relatedness effects in younger but not older adults. Similarly, Miklashevsky et al. (2024) found that motor-relatedness affected lexical decision performance in young adults but not older adults, with no effect on reading aloud in either group. Bidet-Ildei et al. (2020) used a priming paradigm and found that older participants had slightly longer judgment latencies in the congruent conditions, although not significantly. There are two possible explanations for these findings. First, tasks such as lexical decision and reading aloud require less semantic access, especially for older adults (Miklashevsky et al., 2024). Second, these tasks may be more cognitively demanding, leading to floor effects in older adults (Rojas et al., 2024). For example, pseudoword recognition in lexical decision tasks may increase cognitive load, making it more challenging for older adults compared to naming tasks (Rojas et al., 2024).

To sum up, the extant literature suggests that global cognition may influence the presence of or modulate the motor-relatedness effect, especially for older adults, who have relatively poorer global cognitive performance than their younger counterparts and greater cognitive heterogeneity among themselves. However, no study has directly tested this issue. The only two studies that examined the role of motor-relatedness in older adults’ lexical retrieval focused on the contrast between old and young (Miklashevsky et al., 2024; Reifegerste et al., 2021) rather than on individual differences in global cognition within older adults. Thus, further research (with improved design) is clearly needed.

## 2. Methods

The present study aimed to (1) investigate the effect of motor-relatedness on object naming performance (indexed by naming latency) in healthy older adults and (2) investigate whether individual characteristics (i.e., age and global cognition) can modulate the effect of motor-relatedness on object naming performance (indexed by naming latency) in healthy older adults. Therefore, we recruited a group of community-dwelling older adults to complete a timed picture-naming task and a Chinese version of the MoCA. We conducted a linear mixed-effects regression analysis, controlling for several potentially confounding stimulus-related factors, i.e., age of acquisition, familiarity, name agreement, and visual complexity, and two participant-related factors, i.e., gender and education. We hypothesized that older adults would be faster at naming highly motor-related objects, and that older adults with older age and weaker global cognition would benefit more from the motor-relatedness effect.

### 2.1. Participants

A total of 300 community-dwelling older adults were initially recruited for this study from Hangzhou, China. All participants completed the Montreal Cognitive Assessment (MoCA) and provided information about their demographic and linguistic background information through structured questionnaires. Based on the inclusion criteria, participants were required to: (1) be native speakers of Mandarin Chinese; (2) have completed education beyond primary school; and (3) have no evidence of severe cognitive impairment (MoCA score ≥ 18, a cutoff chosen to exclude individuals with severe cognitive impairment, while including those with potential mild cognitive changes associated with normal aging; Mellor et al., 2016; Song et al., 2023) or relevant medical history (brain lesions, stroke, Alzheimer’s disease, etc.). Mild cognitive impairment (MCI), characterized by cognitive decline that does not significantly impair daily functioning, is prevalent among community-dwelling older adults and often remains undetected without screening. Given that excluding individuals with MCI would limit the generalizability of findings to this important population, our research aimed to investigate cognitive and motor interactions in a broader, non-clinical context, including individuals with varying levels of cognitive performance. This approach provides a more comprehensive understanding of these interactions in real-world settings. In the end, 76 participants (41 women, 35 men; mean age = 71.3 (SD = 3.73) years, range = 65–81 years) actually completed all the tests in this study. They were in good health, with no significant medical, neurological, or psychiatric conditions, and they reported normal or corrected-to-normal vision and no language impairment. Details of the participants are given in Table 1.

All participants gave informed consent to participate in this study and received a small payment for taking part in the experiment. This study received approval from the Ethics Committee of School of International Studies at Zhejiang University (approval number: SIS2022-03; approval date: 7 November 2022).

### 2.2. Instruments

Two assessment instruments were used in the present study: (1) the Beijing version of the Montreal Cognitive Assessment (MoCA) for global cognition, and (2) picture stimuli with motor-relatedness ratings for object naming.

#### 2.2.1. Montreal Cognitive Assessment (MoCA)

The Beijing version of the MoCA (Nasreddine et al., 2005; available at https://www.mocatest.org/) was used to evaluate global cognition. The MoCA test is a well-validated instrument designed to assess cognitive performance across seven domains: visuospatial/executive functions, naming, memory, attention, language, abstraction, and orientation. The test was administered in a face-to-face setting, with standardized Mandarin audio recordings providing instructions for each task or subtest.

For the current study, the MoCA was also used to screen for severe cognitive impairment. Cut-off scores were adjusted for older Chinese adults based on years of formal education (Mellor et al., 2016; Song et al., 2023). Participants’ MoCA scores ranged from 18 to 30, with a mean score of 24.5 (SD = 2.38), indicating that none of the participants had severe cognitive impairment.

#### 2.2.2. Picture Stimuli with Motor-Relatedness Ratings

The picture stimuli were high-quality colored photographic stimuli of 77 objects from the China Image Set (CIS; Ni et al., 2019). Since pictures with texture and color had higher ecological validity and shortened naming latencies (Ni et al., 2019), we used high-quality pictures to improve participants’ understanding of the naming task (Herrera et al., 2012). For each picture, the CIS database provides empirically tested data for age of acquisition, familiarity, visual complexity, name agreement, etc. We presented the picture stimuli in a pseudorandomized order.

The selection of these 77 images was based on a multi-faceted approach, considering familiarity and visual complexity, rather than focusing solely on motor-relatedness. This approach ensured a balanced representation of objects across various categories, which was essential for the broader objectives of a large-scale research project.

To measure the motor-relatedness of the objects, an independent sample of 20 young native Chinese speakers (14 female, *M*_age_ = 20.8 years, SD_age_ = 0.41, age range = 20–21 years), all naïve to the goal of the ratings, were asked to rate the 77 objects (pictures) in a web-based questionnaire. This sample selection was based on their relatively stable cognitive profiles, which could provide reliable ratings without the interference of age-related factors.

Following Reifegerste et al. (2021), we used a one-to-five scale, where one meant “not associated with body movements” and five meant “strongly associated with one or more specific body movements”. Before applying these ratings to the main study with older adults, we conducted a small-scale comparison to ensure applicability across age groups. This comparison involved a subset of older adults to verify that they understood the rating scale and the concept of motor-relatedness in a similar way to the young raters.

Participants made their judgements according to their knowledge of whether or not the objects were generally associated with body movements. We provided an example and encouraged raters to rate “ping-pong racket” high if they thought it usually involved a particular body movement, even if they had never played ping-pong. None of the raters took part in the experiment proper. In the present study, motor-relatedness ratings were operationalized as a continuous variable (77 objects, *M*_motor-relatedness_ = 3.09, SD_motor-relatedness_ = 0.77, motor-relatedness range = 1.9–4.85). The use of a continuous variable approach was critical, as it could capture the full spectrum of motor relatedness and reduce the potential impact of age-related biases in the analysis. Details of the motor-relatedness rating are provided in Table A1.

### 2.3. Procedure and Coding

This study was conducted in two phases: an initial screening phase and a main testing phase.

Initial Screening Phase: A total of 300 older adults were recruited for the initial screening. During this phase, participants completed the Montreal Cognitive Assessment (MoCA) Beijing version and a series of structured questionnaires to assess their personal and linguistic background. The MoCA Beijing version was administered in a quiet environment to minimize distractions, following the standardized protocol outlined in the MoCA manual, which was applied consistently to all participants. The examiner provided clear instructions and examples for each subtest to ensure comprehension. The assessment was conducted in a one-on-one setting, with the examiner playing recorded audio in Mandarin to provide instructions for each task or subtest. The assessment began with visuospatial/executive tasks, followed by naming, memory, attention, language, abstraction, delayed recall, and orientation. Each section was scored according to the guidelines in the MoCA manual, with a total score of 30. The entire procedure took about 20 min to complete.

Main Testing Phase: After screening, 76 participants who met the inclusion criteria were invited to participate in the main testing phase. This phase included a battery of cognitive and language tests, such as the timed picture-naming test. The main testing session was conducted in a face-to-face setting by trained examiners.

We used the E-Prime 3.0 software for picture stimulus presentation and data collection. The picture stimulus (300 × 300-pixel dimensions) was presented in the center of the screen on a white background. The entire study was conducted in the participants’ first language (e.g., Chinese). Each participant was tested individually in a quiet room. Older adults were instructed to name the objects as quickly as possible while maintaining accuracy. Before the start of the experimental session, the participants carried out three practice rounds to ensure they understood the accuracy criteria before the formal task. They had to speak loudly and clearly without making any other sounds (e.g., no throat clearing, no preparatory sounds such as “er (呃)” and “en (嗯)”). Each experimental trial began with a 500 ms fixation cross followed by the onset of the picture. All pictures were presented in a pseudorandom order. On each trial, the picture stimulus remained on the screen for a maximum of 3 s (3000 ms). The picture disappeared from the screen and a white screen appeared as soon as a vocal response was registered by the voice key; if there was no response, the picture disappeared at the end of the 3000 ms window. The experimenter pressed a button to unlock the next trial when she was satisfied that the older participant was well prepared. There was no feedback on accuracy. The reaction time (RT) was recorded from the onset of presentation of each stimulus.

Naming responses were coded from the E-Prime recording by two trained research assistants and checked by one of the authors. Only the first response to each picture was taken into account (self-corrected naming errors were not accepted). Responses were coded as correct if they matched the expected response (e.g., 西红柿 “xihongshi”, tomato) or a synonym (e.g., 番茄 “fanqie”, tomato); other responses (e.g., 茄子 “qiezi”, eggplant) or superordinates (e.g., fruit or vegetable, or 篮球 “lanqiu”, basketball referred to as its superordinate 球 “qiu”, ball) were coded as incorrect, as were non-responses. Incorrect naming trials (19.63%) were excluded from the naming latency analyses.

### 2.4. Data Analysis

Linear mixed-effects analysis (using the lme4 package in R) was applied to the naming latencies (for correct responses) in the timed picture-naming task. Raw latency values were log-transformed to improve normality (Kuzmina & Weekes, 2017; Miklashevsky et al., 2024; Reifegerste et al., 2021), and the linearity assumption was judged according to skewness, kurtosis, and visual inspection of residual plots. Moreover, multicollinearity has been shown to affect the accuracy of a model (Fox & Monette, 1992). The variance inflation factor (VIF) method was used, with values greater than 5 or 10 indicating severe multicollinearity (O’brien, 2007). We used mixed-effects regression analyses for several reasons. First, linear mixed models can better account for within-subject correlations compared to analysis of variance. Second, regression analysis allowed for motor-relatedness to be treated as a continuous variable. Most importantly, in terms of the interaction hypothesis, using regression in conjunction with a continuous measure of motor-relatedness allowed us to estimate the precise level at which participant-related factors may or may not have an effect on performance.

We used backward elimination with the step () function in R to identify the best-fit model for log-transformed RTs. The step () function automatically calculates the Akaike Information Criterion (AIC) and performs model comparisons, eliminating effects that did not improve the model fit (*p* > 0.05) (Burnham & Anderson, 2004).

In our initial model (i.e., before backward elimination), the fixed part included all predictor variables of interest and covariates. Our predictor variables of interest were motor-relatedness, age, and the MoCA score, and the interaction terms of motor-relatedness by MoCA (i.e., the MoCA score) and motor-relatedness by age. Two participant-related factors (i.e., gender and education) and five stimulus-related factors (i.e., age of acquisition, name agreement, familiarity, visual complexity, sequence <trial position within the experiment>) were used as covariates to control for their potential influence. Sequence was also included to remove residual autocorrelation and to control for trial-level task effects (Reifegerste et al., 2021). We included interactions between each participant-related factor and motor-relatedness in the initial model. Participant and stimulus were used as random factors. Following Barr et al. (2013), we started with a maximal random effects structure and simplified the model in cases of convergence failure. This led to the inclusion of both participants and stimulus as random effects (random intercepts) for the latency analyses.

To validate the selected model, we compared AIC and Bayesian Information Criterion (BIC) values using the anova () function. In addition, to address potential bias in our estimates, we performed a standard nonparametric bootstrap procedure with 1000 resamples to assess the stability and reliability of the statistics of interest (Lubke et al., 2017).

The continuous independent variables were mean-centered and scaled, and the categorical independent variables were assigned sum coding (e.g., −0.5 and 0.5; Barr et al., 2013; Reifegerste et al., 2021). The significance level was set at *p* < 0.05.

## 3. Results

No participants or picture stimuli were excluded from the data set prior to data analysis. We excluded trials with log-transformed RTs exceeding 3 SDs from the per-participant means, resulting in a data loss of 0.21% of the correct naming trials.

In a normal distribution, a skewness close to 0 indicates symmetry (Singh et al., 2019), and values between −1 and 1 are acceptable. The original RT data had a skewness of 1.32, indicating a significant positive skew. After the log transformation, it decreased to 0.42, which is within the acceptable range and indicates an improvement in symmetry. We also analyzed the kurtosis. The original RT data had a kurtosis of 1.71, indicating a more peaked distribution than the normal (kurtosis = 0). After transformation, the kurtosis dropped to 0.11, getting the distribution nearer to normal.

To ensure that our statistical models were reliable, we checked for multicollinearity using the variance inflation factor (VIF). The results indicated that all VIF values were below 2, well within the acceptable range (VIF < 5 is generally considered acceptable, but VIF < 2 indicates negligible multicollinearity; O’brien, 2007). This result confirms the absence of significant multicollinearity, ensuring the stability of the estimates and the credibility of the results.

In the backward elimination process, the key variables removed due to their non-significant contributions (*p* > 0.05) included motor relatedness: age, whose role in the model was extremely insignificant; sequence, which also showed no significant effect; and motor-relatedness: gender, gender, visual complexity, age, and familiarity. The *p*-values of these variables were all greater than 0.05, and their role in the model did not reach a statistically significant level, so they were successively removed from the model.

**Figure 1:**
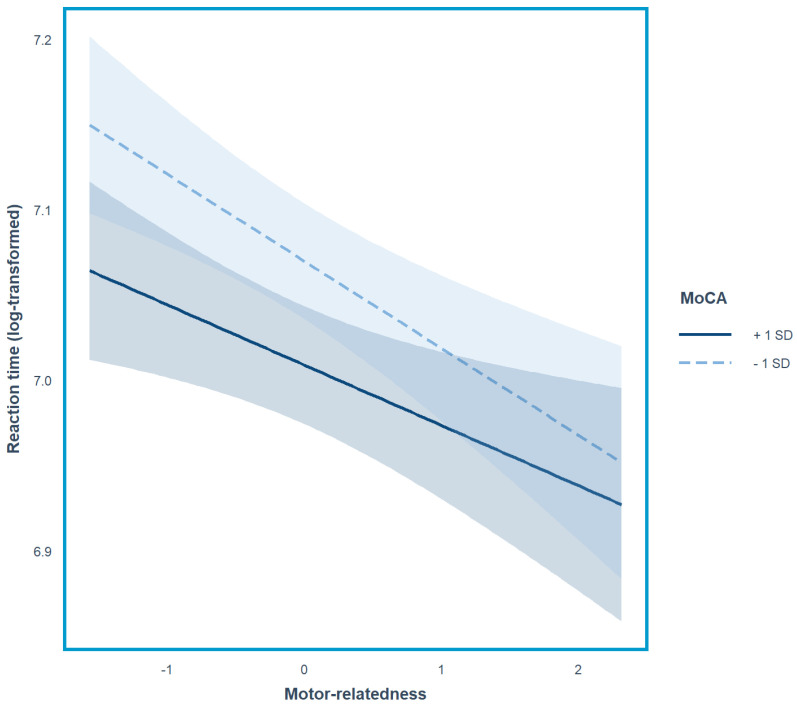
Log-transformed mean RTs, adjusted from the best-fitted model. The model revealed a significant main effect of motor-relatedness and a significant interaction between motor-relatedness and MoCA. Follow-up analyses (simple slope analysis) revealed significant effects of motor-relatedness for older adults with low (−1SD) MoCA scores and a marginally significant effect of motor-relatedness for older adults with high (+1SD) MoCA scores.

Table 2 reports the output (including the estimates and the associated statistics for each fixed effect of interest) of the best-fit linear mixed-effects regression model. The analysis revealed a significant main effect of motor-relatedness (*β* = −0.0433, *SE* = 0.0194, *t* = −2.2348, *p* = 0.025), a significant main effect of the MoCA score (*β* = −0.0308, *SE* = 0.0147, *t* = −2.0900, *p* = 0.037), and a significant interaction between motor-relatedness and the MoCA score (*β* = 0.0079, *SE* = 0.0040, *t* = 2.0035, *p* = 0.045). For the fixed-effect motor-relatedness, the estimate carries a negative sign, indicating longer naming latencies for lowly motor-related than for high-motor-related objects. For the fixed-effect MoCA, the estimate also carries a negative sign, indicating longer naming latency for low-MoCA-score participants. The significant interaction between motor-relatedness and the MoCA score was further examined by computing simple slopes (Régner et al., 2010) for low-MoCA-score (−1SD) and high-MoCA-score (+1SD) participants (Figure 1). This analysis revealed a significant effect of motor-relatedness on object naming latency in low-MoCA-score older adults (*β* = −0.05, *SE* = 0.02, *t* = −2.58, *p* = 0.01) but only a marginal significant effect in high-MoCA-score older adults (*β* = −0.04, *SE* = 0.02, *t* = −1.79, *p* = 0.08).

However, there was no significant main effect of age (*β* = 0.0120, *SE* = 0.0141, *t* = 0.8514, *p* = 0.395) in the initial model and no significant interaction between motor-relatedness and age (*β* = −0.0017, *SE* = 0.0040, *t* = −0.4132, *p* = 0.680), in the initial model (age was eliminated in the best-fit model).

## 4. Discussion

The present study investigated the role of motor-relatedness and its interaction with age and global cognition (evaluated by the MoCA) in lexical processing in older adults. We examined this issue using data from a timed picture-naming task (indexed by naming latency) while controlling for several potentially confounding stimulus-related factors (i.e., age of acquisition, familiarity, name agreement, and visual complexity) and two participant-related factors (i.e., gender and education). The following two predictions were made: (1) older adults would name highly motor-related objects faster; and (2) age and MoCA scores would modulate the motor-relatedness effect, namely that older adults with older age and lower MoCA scores would benefit more from the motor-relatedness effect.

As predicted, our results showed that high motor-relatedness accelerated older adults’ object naming and that older adults with low MoCA scores benefited more from the motor-relatedness effect than older adults with high MoCA scores. The motor-relatedness effect and the modulation effect (between motor-relatedness and MoCA scores) found in our study cannot be explained by the covariates in the initial model (i.e., age of acquisition, familiarity, name agreement, visual complexity, age, gender, and education). In addition, we took motor-relatedness as a continuous variable, avoiding the potential confounding effect of categorization (such as the possible living vs. non-living categorization effect in Reifegerste et al., 2021). In addition, the picture stimuli in our experiment were not specifically chosen to investigate motor-relatedness and may therefore have a high degree of randomness. Furthermore, we did not delete any participants or picture stimuli, and the sample size of 76 participants was relatively large. Therefore, we believe our results were reliable.

Our first finding on the isolated role of motor-relatedness in older adults’ object naming is consistent with the relevant literature on manipulability (Guérard et al., 2015; Lorenzoni et al., 2018) and motor-relatedness (Reifegerste et al., 2021). Reifegerste et al. (2021) found that older adults named motor objects more accurately than non-motor objects. Embodied cognition had the most explanatory power for the motor-relatedness effect found in the present study. Embodied cognition suggests that human cognition is shaped by the human body and environment (Tanton, 2023), and sensorimotor experience helps build strong links between concepts, words, and actions (Glenberg & Gallese, 2012; Negri et al., 2022). Therefore, the link between high-motor-related words and actions may be stronger than for low-motor-related words, and it may be less cognitively demanding to activate or use the action representation (Miklashevsky et al., 2024; Reifegerste et al., 2021). This explanation is consistent with a series of neuroimaging findings. First, there is evidence that viewing or imaging motor-related objects can activate some motor circuits (Dreyer et al., 2015; Klepp et al., 2019; Shtyrov et al., 2014; Tomasino et al., 2012). Second, there is evidence that recruiting motor circuits can facilitate motor-related word retrieval. For example, Branscheidt et al. (2018) found that increasing excitability in the left motor cortex may facilitate the processing of action words but not object words. Additionally, the supplementary motor area (SMA) has been found to be a predictor of word production reaction time (Barragan et al., 2023; Bullock et al., 2023).

Our second finding that global cognition (evaluated by the MoCA) modulated the motor-relatedness effect on object naming in older adults is a novel finding. This finding supports Reifegerste et al.’s (2021) proposal that people begin to massively recruit motor circuitry only when other lexical retrieval mechanisms (e.g., recruiting working memory network and prefrontal scaffolding; Crowther & Martin, 2014; Park & Reuter-Lorenz, 2009) weakened. In other words, although recruiting motor circuitry may facilitate word processing (as discussed in the previous paragraph), high-motor-related words do not “obligatorily” recruit motor circuitry in young adults and high-MoCA-score older adults (Montero-Melis et al., 2022; Zeelenberg & Pecher, 2016).

First, this explanation is consistent with the scaffolding theory of aging and cognition (STAC) in Park and Reuter-Lorenz (2009) and Cotelli et al. (2012). STAC predicts that older adults recruit compensatory brain circuitry (scaffolds) to maintain adequate task performance and that scaffolding processes result in greater bilateral activation and overactivation, typically in prefrontal areas (Park & Reuter-Lorenz, 2009; Cotelli et al., 2012). Although there is no direct evidence on the role of prefrontal areas in predicting global cognition (i.e., MoCA scores), Nosaka et al. (2022) found that prefrontal oxygenated hemoglobin levels were significantly lower in low-MoCA-score than in high-MoCA-score older adults during dual-task walking. Dual-task walking is a walking task in which an additional cognitive task is added during walking. In this study, a letter fluency task was added during dual-task walking, in which acronyms (e.g., those beginning with “a”) had to be recalled. Their results suggest that high-MoCA-score participants may use a compensatory neural strategy (i.e., overactivation in the prefrontal cortex) to maintain their gait speed. Similarly, high-MoCA-score participants in our experiment may have adopted this neural strategy (i.e., reduced lateralization and overactivation in prefrontal areas) to avoid lexical retrieval failure.

Second, the finding that low-MoCA-score older adults benefit more from the motor-relatedness effect suggests that motor circuitry may provide a complementary approach to compensate for cognitive aging (evaluated by the MoCA) and helps refine and advance the scaffolding theory of aging and cognition (STAC). As the prefrontal cortex of low-MoCA-score participants may have deteriorated to the extent that prefrontal scaffolding cannot support them to successfully retrieve the object name within 3000 ms (the time window), they recruited other scaffolding circuits (e.g., motor circuits). Importantly, our results show that motor-relatedness (*β* = −0.0433) is more efficient than MoCA scores (*β* = −0.0308) in facilitating older adults’ lexical retrieval (see Table 2), which is consistent with STAC’s prediction and previous evidence (see Wierenga et al., 2008). STAC predicted that prefrontal scaffolding (i.e., activation of a larger prefrontal network) would be less efficient than the specific network in the younger brain (as reflected by longer naming latency with equivalent accuracy, Wierenga et al., 2008; see also Park & Reuter-Lorenz, 2009) but may at least help maintain adequate performance. In other words, when the task is familiar, scaffolding would not be invoked (e.g., young adults naming objects), but when a familiar task becomes challenging, people with higher global cognition (i.e., young adults and older adults with high MoCA) may only scaffold in prefrontal areas, e.g., naming objects in a less proficient second language (Hernandez & Meschyan, 2006), naming objects depicted from unusual viewpoints (Gigi et al., 2007), and older adults naming objects under time pressure (our experiment). However, if older adults’ global cognition has weakened to the extent that overall task performance is challenged, they may begin to massively recruit motor circuits when processing highly motor-related words and thus benefit more from the motor-relatedness effect. Therefore, our finding that people with low MoCA scores gain more benefits from the motor-relatedness effect may help explain the mixed results in the previous literature (e.g., Guérard et al., 2015; Lorenzoni et al., 2018; Ni et al., 2019).

Additionally, in our investigation, age had neither a main effect nor an interaction with motor-relatedness on object naming, which is different from Reifegerste et al.’s (2021) investigation. A possible explanation for the difference is that Reifegerste et al. (2021) contrasted the difference between the old and the young (*M*_age_ = 41.8 years, SD_age_ = 17.3, age range = 18–72), whereas our study focused on the difference within older adults (*M*_age_ = 71.3 years, SD_age_ = 3.73, age range = 65–81). Young adults may have reached a peak in global cognition during their lifetime, whereas older adults experience cognitive decline to varying degrees. Therefore, the age effect and the interaction of age with motor-relatedness found in Reifegerste et al. (2021) could be due to differences in global cognition (measured by the MoCA) between the young and the old. However, the decline in global cognition is not exactly in line with aging, and there are large individual differences in global cognition among older adults. Our research suggests that global cognition (evaluated by the MoCA) is a better predictor of object naming latency than age.

Although neither stimulus- nor participant-related covariates were of interest in our investigation, some of them are worth summarizing here. First, the significant main effects of age of acquisition and name agreement (both with a positive sign of the estimate, see Table 2), the marginally significant main effect of familiarity (with a negative sign of the estimate), and no significant effect of visual complexity were observed. These results are in line with previous studies (e.g., Guérard et al., 2015; Lorenzoni et al., 2018; Reifegerste et al., 2021). Second, in addition to the two factors of interest presented in the Results Section, we examined education and gender as covariates. A marginally significant main effect of education was observed (with a negative sign of the estimate, see Table 2) and a significant interaction between education and motor-relatedness. This observation does not yet have a strong explanation, but it adds to the literature in this area. Thirdly, no main effect of gender and no interaction between gender and motor-relatedness was observed in our experiment.

This study has three limitations. First, it focused on a specific age range of older adults (65–81 years old), which may be part of the reasons for the non-significance of age-related effects on naming latency. Second, certain object stimuli used in the study (e.g., computer) may be very familiar to younger people due to regular use, whereas older adults may not be accustomed to these items, which could lead to a bias in the rating of motor-relatedness. Third, the general linear mixed-effects analysis revealed that older adults exhibited greater accuracy in naming high-motor-related objects, but this finding did not reach statistical significance. This may be due to the fact that the experiment was not designed to assess accuracy. Responses elicited by noise, throat clearing, or coughing were coded as incorrect, and those longer than 3000 ms were not recorded. In addition, the stimulus-related covariates were from the China Image Set (CIS; Ni et al., 2019), which focuses on naming latency rather than accuracy. These factors likely influenced the accuracy analysis and should be addressed in future research.

The present study has several implications. First, it investigated the motor–language association in healthy older adults and found that global cognition can modulate the motor-relatedness effect, helping explain the conflicting results found in young adults; accordingly, it provides empirical evidence from older adults so as to better explain embodied cognition from the perspective of lifelong development. Second, it shows that older adults’ motor circuitry may provide a complementary approach to compensate for cognitive ageing (measured by the MoCA) and helps to refine and advance the scaffolding theory of ageing and cognition (STAC) by demonstrating the flexibility of motor circuitry. Third, it provides evidence that motor-relatedness (a plastic factor associated with one’s own motor experience) facilitates lexical retrieval in older adults and helps to identify which type of population (i.e., low-MoCA older adults) will be more sensitive to the motor intervention; researchers are encouraged to provide older adults with motor-related linguistic interventions. For example, new intervention protocols can be designed to strengthen the association between a low-motor-related word (e.g., mountain) and a relevant action (e.g., climbing a mountain; Reifegerste et al., 2021).

In conclusion, the present study investigated two issues that have not been explored in the literature up to date (as far as we know) and had two major findings. First, we found that older adults name objects with high motor-relatedness faster, which parallels a number of previous findings in the literature at both the behavioral and neural levels. Second, we found that low-MoCA-score older adults benefit more from the motor-relatedness effect and compensate for their degraded cognition to maintain a relatively intact lexical retrieval ability, which has important research and application values.

## Figures and Tables

**Table 1:** Demographic information and global cognition (MoCA) scores.

	N (%)	Mean (SD)	Range
Gender			
Female	41 (53.9%)		
Male	35 (46.1%)		
Age (years)		71.3 (3.73)	65–81
Education (years)		9.80 (2.72)	6–18
MoCA score (0–30)		24.5 (2.38)	18–30

**Table 2:** Model output from the best-fit linear mixed-effects regression model.

Random Effects	Name	Variance	SD	
Picture stimulus	(Intercept)	0.0241	0.1553	
Participant	(Intercept)	0.0132	0.1147	
Residual		0.0628	0.2506	
**Fixed Effects**	***β***	***SE***	***t*-Value**	***p*-Value**
(Intercept)	7.0526	0.0224	314.8365	<0.001
Motor-relatedness	−0.0433	0.0194	−2.2348	0.025
Education	−0.0260	0.0147	−1.7652	0.078
MoCA	−0.0308	0.0147	−2.0900	0.037
Age of acquisition	0.1168	0.0204	5.7282	<0.001
Name agreement	0.0559	0.0191	2.9235	0.003
Motor-relatedness: education	−0.0084	0.0038	−2.1925	0.028
Motor-relatedness: MoCA	0.0079	0.0040	2.0035	0.045

## Data Availability

The experimental materials, analysis code, and data generated and analyzed during this study are available from the corresponding author upon reasonable request.

## Appendix A

**Table A1:** Object names and the motor-relatedness rating.

Object Names (Chinese)	Object Names (English)	Motor-Relatedness Rating	Object Names (Chinese)	Object Names (English)	Motor-Relatedness Rating
白菜	Napa cabbage	2.35	芒果	Mango	2.3
包菜	Wild cabbage	2.25	毛笔	Ink brush	3.95
笔记本	Notebook	3.15	奶牛	Dairy Cow	2.65
玻璃杯	Glass	3.05	南瓜	Pumpkin	2.25
菠萝	Pineapple	2.35	镊子	Tweezers	4.1
菜刀	Kitchen knife	4.05	喷雾器	Sprayer	3.8
草莓	Strawberry	2.25	乒乓球拍	Table tennis racket	4.85
叉子	Fork	3.85	苹果	Apple	2.6
插座	Sockets	2.85	七星瓢虫	Seven-spot ladybird	1.95
衬衫	Shirt	3	青椒	Green pepper	2.3
橙子	Orange	2.3	热水器	Electric water heater	2.6
抽油烟机	Exhaust hood	2.9	三角尺	Trianglar ruler	3.1
打火机	Lighter	3.6	三轮车	Tricycle	3.6
大巴	Bus	3.35	色子	Dice	3.7
凳子	Stool	2.7	沙发	Sofa	2.9
电话	Telephone	3.4	鼠标	Mouse	3.85
电脑	Computer	3.45	刷子	Brush	4.3
电暖气	Electric heater	2.65	算盘	Abacus	4.2
电熨斗	Clothes iron	4.15	太阳能	Solar water heater	2.3
冬瓜	Wax gourd	2.2	土豆	Potato	2.3
放大镜	Magnifying glass	3.55	碗	Bowl	2.95
钢琴	Piano	4.2	微波炉	Microwave	3.2
鸽子	Pigeon	2.2	西瓜	Watermelon	2.65
公鸡	Rooster	2.35	洗衣机	Washing machine	2.85
鼓	Drum	4.1	香菜	Corriander	2.45
核桃	Walnut	2.55	香烟	Cigarette	3.35
蝴蝶	Butterfly	1.9	向日葵	Sunflower	1.95
夹子	Tongs	3.95	橡皮	Eraser	3.55
剪刀	Scissors	4.05	小号	Trumpet	4.1
节能灯	Energy-saving light	2.65	小提琴	Violin	4.5
镜子	Mirror	2.7	靴子	Boot	3.95
卷尺	Tape measure	3.8	鸭子	Duck	2.4
篮球	Basketball	4.5	洋葱	Onion	2.3
篮子	Basket	3.6	衣柜	Wardrobe	2.85
老虎	Tiger	2.35	羽毛球	Badminton	4.5
轮船	Ship	3	长颈鹿	Giraffe	1.9
萝卜	Radish	2.35	猪	Pig	2.4
马	Horse	3	桌子	Desk	2.9
马甲	Waistcoat	3.1			
